# Urinary Metabolomic Changes and Potential Exercise-Induced Muscle Damage Biomarkers Identification in Trained Young Males Following Acute Intermittent Rowing Training

**DOI:** 10.3390/bios15120803

**Published:** 2025-12-08

**Authors:** Yang Cheng, Yue Yi, Xuefeng Shi, Shumin Bo

**Affiliations:** 1 School of Life Science, Beijing Institute of Technology, Beijing 100081, China; chengyang2023@cupes.edu.cn; 2 School of Kinesiology and Health, Capital University of Physical Education and Sports, Beijing 100191, China; boshumin@163.com; 3 Department of High Altitude Medicine, Qinghai Provincial People’s Hospital, Xining 810007, China; shixuefeng128@163.com

**Keywords:** exercise-induced muscle damage, urinary biomarkers, metabolomics, rowing training, delayed onset muscle soreness

## Abstract

(1) Background: This study aims to explore the changes in urinary metabolomic profile among trained young males following acute intermittent rowing training (AIRT), and to identify potential urinary biomarkers associated with exercise-induced muscle damage (EIMD). (2) Methods: 22 trained young males were recruited to perform AIRT. The changes in blood biochemical indexes associated with EIMD were analyzed. EIMD occurrence was evaluated using blood biochemical indexes, muscle function, and pain assessment. The changes in urinary metabolites were determined using untargeted metabolomic analysis. (3) Results: Four blood biochemical indices, including creatine kinase, lactate dehydrogenase, creatine kinase-MB, and hydroxybutyrate dehydrogenase, were significantly elevated immediately after AIRT. Furthermore, an obvious immune response appeared, and countermovement jump performance significantly decreased. Among 384 urinary metabolites, 33 were significantly upregulated, and 12 were downregulated immediately after AIRT. Upregulated metabolites were mainly involved in phenylacetate metabolism, ammonia recycling, the urea cycle, and glutathione metabolism. Four potential urinary biomarkers were identified, including 2′-Deoxycytidine, cytosine, Phenylacetaldehyde, and Pyridoxamine. (4) Conclusions: AIRT induced EIMD in all participants and significantly altered urinary metabolite profiles. The changes in urinary metabolites and pathways were due to the metabolic adaptation to oxidative stress, inflammatory responses, and ammonia metabolism imbalance. The selected four potential urinary biomarkers provide important evidence for the further development of a non-invasive, urine-based method for the immediate assessment of EIMD.

## 1. Introduction

Athletes in competitive sports often face the challenge of exercise-induced muscle damage (EIMD) [1]. EIMD refers to microstructural damage to muscle fibers and cellular structures caused by excessive or high-intensity exercise loads, especially during unaccustomed exercise [2]. Despite that EIMD generally disappears within a few days, it may also trigger a series of physiological stress responses, including metabolic dysregulation [3], immunological stress [4], and oxidative stress [5]. Additionally, EIMD may lead to decreased muscle function, increased risk of injury, and even induce rhabdomyolysis, ultimately affecting athletic performance and having adverse effects on overall health [6,7]. Therefore, diagnosis and early prediction of EIMD are important to ensure athlete health.

EIMD is often externally manifested as delayed onset muscle soreness (DOMS). However, DOMS occurs ~24 h after exercise and cannot provide timely information for the early assessment of EIMD [8]. While muscle biopsy remains the most accurate method for diagnosing EIMD, it may cause localized tissue damage [9]. Blood biomarkers, e.g., creatine kinase (CK) and lactate dehydrogenase (LDH), exhibit high sensitivity and are widely used to evaluate EIMD [10]. Furthermore, creatine kinase-MB isoenzyme (CK-MB) and hydroxybutyrate dehydrogenase (HBDH) are selected to assist in the assessment of EIMD [11]. Several indicators reflecting inflammatory response are also used in the analysis of EIMD [12]. However, these indicators are invasive, and frequent sampling may disrupt athletes’ training regimens [13]. Recently, imaging techniques such as magnetic resonance imaging [14] and computed tomography [15] provide alternative non-invasive methods, but these methods rely on large and expensive imaging equipment, which are not suitable for on-site analysis.

In addition to blood biochemical indicators, urinary indices are also related to physiological stress responses. Urine specific gravity, a key indicator of hydration status and fluid loss, has been widely used in monitoring hydration levels [16]. Meanwhile, urea nitrogen is commonly used to indicate enhanced protein breakdown [17]. The main reason is that prolonged high-intensity exercise leads to substantial glycogen depletion, prompting the body to increasingly rely on protein catabolism for energy. In addition, urinary protein and occult blood are often used to evaluate the degree of fatigue and adaptation to physical load [18,19]. However, specific urinary biomarkers for EIMD have not yet been identified or studied.

So far, a series of studies have utilized metabolomics to investigate the dynamic changes in urinary metabolites before and after exercise. For instance, Wang et al. used ultra-high-performance liquid chromatography–mass spectrometry to analyze the urinary metabolic changes in female athletes after high-intensity water polo training, identifying 27 differential metabolites and indicating that the nicotinic acid and nicotinamide metabolic pathways play a key role in post-exercise urinary metabolism [20]. Zhao et al. revealed the impact of high-intensity interval training (HIIT) on steroid hormone metabolites and amino acid biosynthesis in young soccer players, providing a theoretical foundation for the molecular mechanisms underlying HIIT-induced long-term adaptations [21]. In addition, Schranner et al. found that approximately 196 metabolites exhibited significant changes within 24 h post-exercise [22]. Kistner et al. differentiated the effects of two endurance exercise intensities on urinary metabolites and metabolic pathways [23]. However, all these studies did not systematically evaluate the occurrence of EIMD, thus failing to explore the correlation between specific metabolites and EIMD. To date, only one study has preliminarily investigated changes in the urinary metabolome after EIMD [24]. However, these metabolite changes occurred 24 h after exercise and cannot support the early assessment of EIMD.

Therefore, this study aims to investigate the urinary metabolomic profile in trained young males following acute intermittent rowing training (AIRT) and to identify potential urinary biomarkers associated with EIMD. First, participants were recruited to perform an AIRT protocol, and both blood and urine samples were collected before and after training. Then, blood samples were analyzed to evaluate muscle damage and immune stress, while urine samples were subjected to untargeted metabolomic profiling. Additionally, muscle function and perceived soreness were assessed. Finally, potential urinary biomarkers associated with EIMD were screened.

## 2. Materials and Methods

### 2.1. Participants

A total of 22 young male participants with a background in athletic training were recruited. All participants were aged between 18 and 25 years, with an average training experience of 6.39 ± 3.13 years. All the participants were in good health, had not experienced any recent infections, had not received medication (such as antibiotics, steroids, or anti-inflammatory drugs), and had not used any vitamins or other supplements. Furthermore, all the participants also had no history of muscle injuries, sprains, fractures, or other sports-related injuries in the recent past. The participants’ basic characteristics are shown in Table 1.

### 2.2. Exercise Protocol

All the participants were asked to perform rowing training using a magnetic resistance rowing machine (MRH3208A, Mobifitness Co., Shanghai, China) (laboratory environment: room temperature 24 °C, humidity 37%). The rowing distance was 3 km, and the resistance was set at the maximum level of 30 (more than 85% of maximal heart rate), resulting in approximately 600 rowing strokes. After every 200 m of rowing, 30 s of intermittent active recovery was provided by changing the resistance to 1. To ensure training efficiency and safety, heart rate was continuously monitored in real time using a Polar heart rate monitor (Polar H10, Polar Electro, Kempele, Finland).

### 2.3. Post-Exercise Muscle Function Assessment

Lower limb muscle function was evaluated by using a countermovement jump (CMJ) measuring device (ZTJ-II Countermovement jump Tester, Xindong Huateng Sports Equipment Co., Beijing, China) before and immediately after exercise (within 5 min). All participants wore sports shoes, and each participant was asked to perform a 5 min self-directed warm-up before the CMJ test. Each test was performed in triplicate. At 24 and 48 h after exercise, DOMS was assessed using a VAS to quantify soreness [25]. The VAS ranges from 0 to 10, where 0 indicates no pain and 10 represents extreme pain.

### 2.4. Blood Biochemical Analysis

Venous blood samples were collected before exercise (following a standardized breakfast and approximately 1 h of rest) and immediately after exercise (within 5 min) for each participant. The biochemical indices related to muscle damage and immune response were analyzed. Blood biochemical indexes related to muscle damage, including CK, CK-MB, LDH, and HBDH, were tested before and immediately after the exercise. Immune indexes, including leukocyte count, neutrophil count, monocyte count, lymphocyte count, and platelet count, were also analyzed. All the tests were accomplished with the assistance of Beijing Institute of Technology Hospital. Paired sample *t*-tests were performed for significant difference analysis using Prism software (version 10.2, GraphPad Software, Boston, MA, USA), with the significance level set at *p* < 0.05.

### 2.5. Urinary Metabolomics Analysis

Participants were required to abstain from water intake prior to urine collection, and midstream urine samples were collected before and immediately after exercise (within 5 min) for urine metabolomics analysis. Specifically, urinary metabolites were revealed by using LC-MS and were identified by comparing the spectrum data to a high-quality tandem mass spectrometry database. To ensure accuracy, only metabolites with a coefficient of variance below 30% in quality control samples were retained. For metabolite quantification, chromatographic peaks detected in the samples were integrated using XCMS software (version 4.0, The Scripps Research Institute, La Jolla, CA, USA), with the area of each peak representing the relative abundance of the corresponding metabolite. In this study, QC samples were prepared by mixing all the experiment samples. QC samples were analyzed in triplicate.

Non-endogenous metabolites were excluded by using the HMDB database, and the remaining metabolites were normalized using creatinine. The metabolic trends of urine samples before and after exercise were visualized through principal component analysis (PCA) and cluster heatmaps. Paired t-tests were conducted to analyze differential metabolites, with a significance level set at 0.05. Metabolic pathways enriched by differential metabolites were analyzed using the HMDB database. Orthogonal partial least squares discriminant analysis (OPLS-DA) and correlation analysis were applied to identify potential urinary biomarkers for EIMD. The diagnostic performance of these potential biomarkers was evaluated using the area under the receiver operating characteristic curve (ROC). The selection of biomarkers was mainly based on the following two criteria: absolute value of correlation coefficient (R) > 0.3, and a variable importance in projection (VIP) value > 1.

## 3. Results

### 3.1. Blood Indicators and EIMD

**Figure 1:**
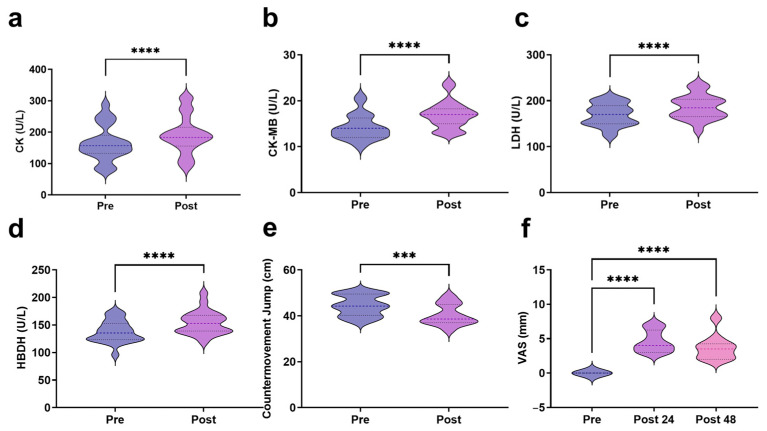
Blood markers and muscle function indicators related to EIMD ((**a**): CK; (**b**): CK-MB; (**c**): LDH; (**d**): HBDH; (**e**): CMJ; (**f**): VAS). *** indicates *p* < 0.001, and **** indicates *p* < 0.0001.

All the participants finished the rowing exercise. The results demonstrated a significant increase in serum CK levels following exercise, rising from 164.14 ± 56.24 U/L at baseline to 191.73 ± 60.59 U/L post-exercise (t = 9.176, *p* < 0.0001) (Figure 1a). CK-MB levels exhibited a marked elevation from 14.23 ± 2.79 U/L to 16.95 ± 2.95 U/L (t = 5.134, *p* < 0.0001) (Figure 1b). LDH significantly increased from 169.32 ± 24.38 U/L to 186.32 ± 26.56 U/L (t = 7.997, *p* < 0.0001) (Figure 1c). HBDH levels showed a significant post-exercise rise from 138.00 ± 19.81 U/L to 154.05 ± 21.33 U/L (t = 8.832, *p* < 0.0001) (Figure 1d). CMJ height significantly declined from 44.45 ± 4.47 cm at baseline to 40.38 ± 4.55 cm post-exercise, indicating a decline of 9.15% (t = 4.510, *p* < 0.001) (Figure 1e). DOMS severity was recorded for two consecutive days post-exercise. The results indicated a significant increase in DOMS scores at 24 h post-exercise, which remained significantly elevated at 48 h compared to the baseline (Figure 1f).

### 3.2. Immune Stress Response Following AIRT

**Figure 2:**
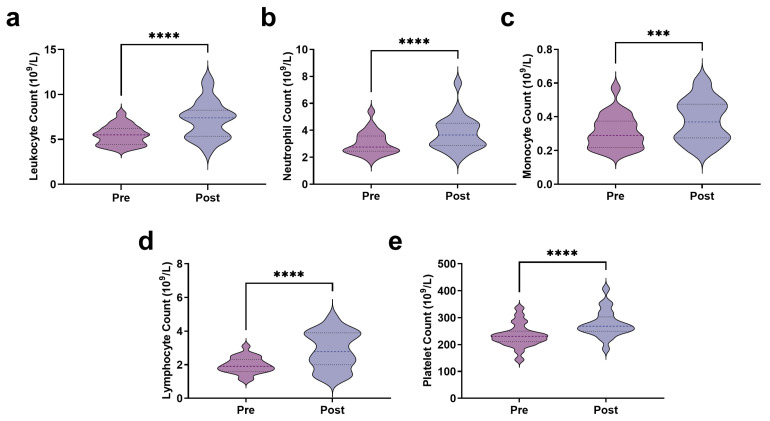
Immune stress response to AIRT ((**a**): Leukocyte count; (**b**): Neutrophil count; (**c**): Monocyte count; (**d**): Lymphocyte count; (**e**): Platelet count). *** indicates *p* < 0.001, and **** indicates *p* < 0.0001.

The results indicate that at rest, the leukocyte count was 5.47 ± 1.07 × 10^9^/L, which significantly increased immediately post-exercise to 7.19 ± 2.07 × 10^9^/L (t = 6.719, *p* < 0.0001) (Figure 2a). Neutrophil counts increased significantly from 3.04 ± 0.86 × 10^9^/L pre-exercise to 3.77 ± 1.23 × 10^9^/L post-exercise (t = 6.077, *p* < 0.0001) (Figure 2b). Similarly, monocyte counts increased from 0.31 ± 0.10 × 10^9^/L pre-exercise to 0.38 ± 0.13 × 10^9^/L after exercise (t = 3.989, *p* < 0.001) (Figure 2c). In addition, lymphocyte counts increased significantly from 1.97 ± 0.48 × 10^9^/L pre-exercise to 2.86 ± 1.09 × 10^9^/L post-exercise (t = 4.991, *p* < 0.0001) (Figure 2d). Notably, AIRT not only affected leukocyte counts but also had a significant impact on platelet levels (Figure 2e). The platelet count increased from 233.82 ± 43.38 × 10^9^/L pre-exercise to 277.27 ± 49.52 × 10^9^/L post-exercise (t = 9.672, *p* < 0.0001), representing an 18.58% increase.

### 3.3. Metabolomics Analysis Results

#### 3.3.1. Overall Trend of Urinary Metabolites Following AIRT

**Figure 3:**
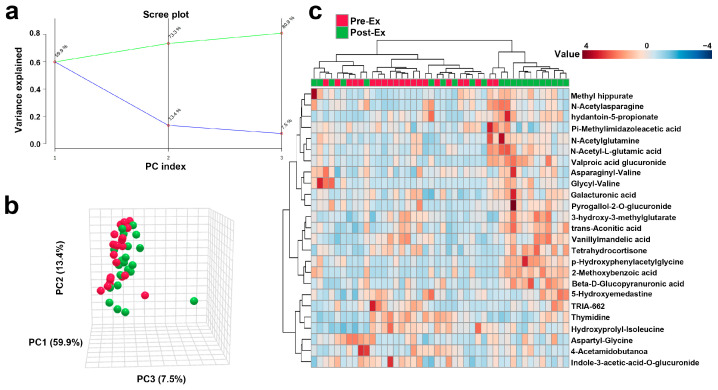
Overall trends in urinary metabolites after AIRT (**a**): The cumulative variance explained by principal components (green line) and proportion of variance explained by each individual principal component (blue line); (**b**): Changes in urinary metabolites after exercise using PCA analysis (Red spheres represent samples of Pre-Ex, and green spheres represent samples of Post-Ex); (**c**): Cluster heatmap.

The PCA results indicate that the cumulative contribution of PC1, PC2, and PC3 reached 80.8% (Figure 3a), indicating that more than 80% variation is explained by the three principles. The two groups, with red and green dots representing pre- and post-exercise samples, were separated in a three-dimensional PCA plot (Figure 3b). Forty-four samples were clustered into two groups. The left sub-cluster mainly included samples before exercise, while the right sub-cluster mainly included samples after exercise. The clustering achieved an accuracy of 0.727, indicating a reasonably good match between the predicted clusters and the true class labels (Figure 3c).

#### 3.3.2. Changes in Urinary Metabolites and Metabolic Pathways After AIRT

**Figure 4:**
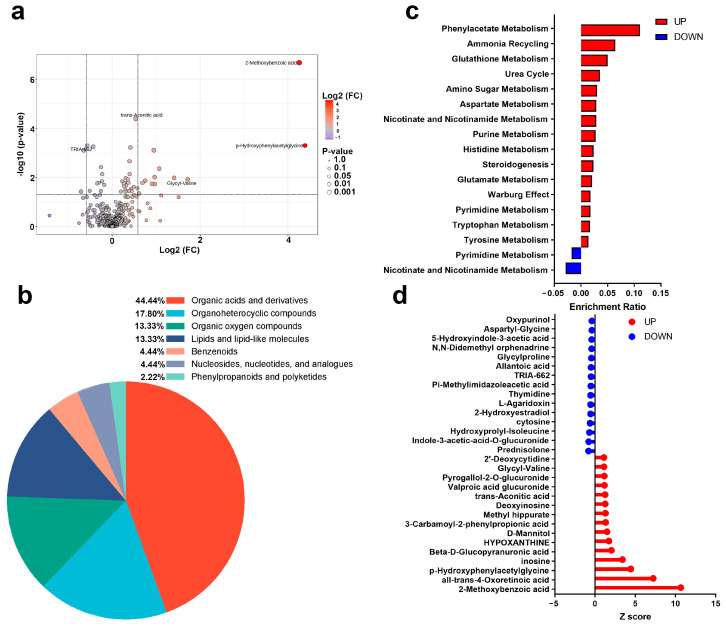
Post-AIRT urinary metabolites and metabolic pathways ((**a**): Volcano plot; (**b**): Metabolite annotation; (**c**): Metabolic pathways; (**d**): Z score).

A total of 45 urinary metabolites exhibited significant alterations following AIRT. To visualize these changes, a volcano plot was constructed using a fold change threshold of 1.5 (Figure 4a). These metabolites predominantly belonged to organic acids and derivatives (44.44%), organoheterocyclic compounds (17.80%), organic oxygen compounds (13.33%), and lipids and lipid-like molecules (13.33%) (Figure 4b). The upregulated metabolites were enriched in 15 pathways, e.g., phenylacetate metabolism, ammonia recycling, and glutathione metabolism pathways. The downregulated metabolites were enriched in pyrimidine metabolism and nicotinate and nicotinamide metabolism (Figure 4c). The Z-score of these differential metabolites ranged from −0.394 to 10.654 (Figure 4d).

#### 3.3.3. OPLS-DA and ROC Analysis Results

**Figure 5:**
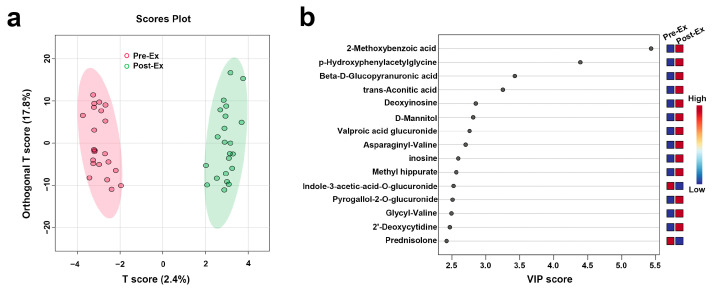
Key metabolites identified for differentiating pre- and post-AIRT samples ((**a**): OPLS-DA; (**b**): VIP Scores).

**Figure 6:**
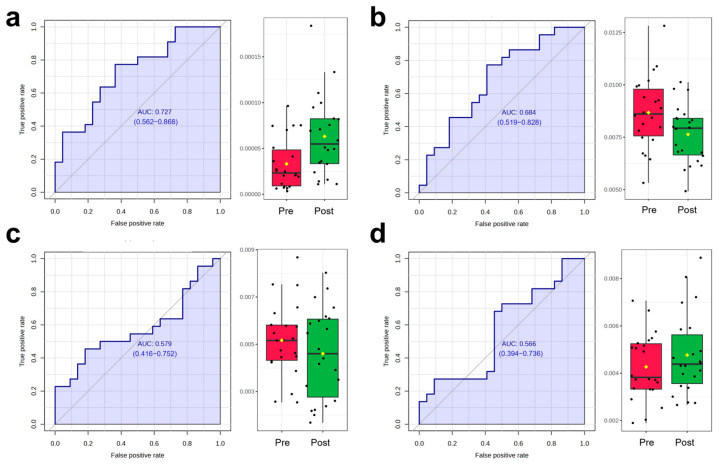
ROC analysis and dynamic changes in the selected metabolites before and after exercise ((**a**): 2′-Deoxycytidine; (**b**): cytosine; (**c**): Pyridoxamine; (**d**): Phenylacetaldehyde) (Black dots represent individual data points/observations, and yellow diamonds represent the mean values).

The scores plot of OPLS-DA is presented in Figure 5a, in which the clear separation between pre-exercise (red) and post-exercise (green) data points along the T score axis was observed. To further identify metabolites contributing to group differentiation, VIP scores were calculated. The top 15 metabolites with high VIP scores included 2-Methoxybenzoic acid, p-Hydroxyphenylacetylglycine, Beta-D-Glucopyranuronic acid, trans-Aconitic acid, Deoxyinosine, D-Mannitol, Valproic acid glucuronide, Asparaginyl-Valine, Inosine, Methyl Hippurate, Indole-3-acetic-acid-O-glucuronide, Pyrogallol-2-O-glucuronide, Glycyl-Valine, 2′-Deoxycytidine, and Prednisolone. To validate the reliability of the model, 2000 permutation tests were performed, yielding a Q^2^ value of 0.573 and an R^2^ value of 0.981, indicating that the model exhibited strong predictive ability and goodness of fit in distinguishing samples before and after AIRT intervention. Subsequently, four metabolites were further identified through correlation analysis and VIP scores, including 2′-Deoxycytidine, Cytosine, Pyridoxamine, and Phenylacetaldehyde, whose variation patterns closely resembled those of CK and LDH (Figure 6) (Table 2). The best prediction performance was obtained using 2′-Deoxycytidine (AUC = 0.727), and the sensitivity and specificity achieved 77.3% and 63.6%, respectively.

## 4. Discussion

Our results indicate a significant increase in CK and LDH levels immediately after AIRT. Similar phenomena have been revealed in recent studies on EIMD. Gomes et al. showed a significant increase in CK immediately after high-intensity functional training (from 174.9 to 226.7 U/L) [26]. Bernat-Ad ell et al. also demonstrated that CK levels significantly increased immediately after a marathon (from 158.48 ± 77.71 U/L to 396.74 ± 244.94) [27]. Similarly, a study by Vilar et al. showed that after 21 h of ultra-endurance running, the LDH levels significantly increased immediately post-exercise, from 190.4 ± 5.9 U/L to 332.5 ± 23.2 U/L [28]. Pokora et al. demonstrated that blood CK levels were significantly elevated in healthy young men following downhill running [29]. Similarly, Hammouda et al. reported that maximal cycling sprints led to significant increases in blood CK and LDH levels in soccer players [30]. Consistently, our study observed a similar immediate elevation, indicating that our training protocol is comparable to these exercise modalities in inducing EIMD. All these studies reported EIMD occurrence after exercise. Therefore, increased CK and LDH levels indicated the appearance of EIMD in this study. A significant increase in CK-MB further illustrated EIMD occurrence. D’Alleva et al. reported a significant increase in CK-MB levels (3.41 ± 3.01 ng/mL) immediately after prolonged exercise in trained individuals [31].

A significant decrease in CMJ performance and DOMS appearance after AIRT further indicated EIMD occurrence [32,33]. Heileson et al. found a significant decrease in CMJ performance immediately following downhill running and jumping lunges [34]. Additionally, Rosvoglou et al. used an isokinetic dynamometer to induce muscle damage and found significantly reduced CMJ performance immediately post-exercise [33]. DOMS appeared 24 h post-exercise and remained even 48 h after exercise. A similar phenomenon was also observed in the study by Chen et al., in which VAS also increased at 24 and 48 h post-exercise [35].

Muscle damage and repair in EIMD rely on the immune response. Our study shows that immediately after AIRT, leukocyte counts increased by approximately 31.26%. Similarly, a study by Gomes indicated a significant 38% increase in leukocyte counts after a sprint race, in which muscle damage was observed following the race [36]. A significant increase in neutrophils and monocytes immediately after AIRT was also observed, which is associated with the mobilization of immature neutrophils [37]. During exercise-induced acute inflammation, neutrophils and monocytes are the first responders, regulating the initial immune response [38]. Lymphocytes also exhibit an initial increase after exercise [39]. The significant increase in lymphocytes immediately after acute exercise is likely attributed to a several-fold increase in the number of CD8+ T and CD4+ T cells [40]. Therefore, all these increased immune responses further demonstrated EIMD occurrence.

In the present study, a total of 45 urinary metabolites showed significant changes after exercise, with 33 upregulated and 12 downregulated. Among these differential metabolites, some have been reported in previous studies. For instance, Mannitol was significantly elevated post-exercise in this study, which is consistent with Kistner et al., who reported a significant increase in Mannitol following a standardized exercise tolerance test [41]. However, some metabolites exhibited different trends compared to previous studies. For example, Glutamine levels increased in the present study, which is in line with observations after running [42], but no change was observed following acute resistance exercise [43], possibly due to differences in exercise protocols. In fact, most of the differential metabolites identified have not been reported previously. Therefore, this study expands our understanding of urinary metabolite alterations following high-intensity exercise.

Our study found the significant upregulation of glutamate metabolism, along with the increase in phenylacetate, glutathione, nicotinate, and nicotinamide metabolism and ammonia recycling pathways, which collectively mitigate the oxidative stress induced by AIRT, thereby facilitating muscle cell repair and functional recovery [44]. Similarly, a significant increase in aspartate metabolism was observed post-AIRT, promoting urea cycle activity to efficiently eliminate excess ammonia [45]. In addition, purine metabolism upregulation may be due to the accelerated nucleic acid degradation following EIMD [46]. The upregulation of amino glycan metabolism mainly supports cell membrane repair and matrix remodeling, further accelerating the repair of muscle damage [47]. All these upregulated metabolic pathways reflect a complex metabolic adaptation process by the body to address EIMD, oxidative stress, inflammation, and nitrogen metabolism imbalance.

In this study, four metabolites, including 2′-Deoxycytidine, Cytosine, Pyridoxamine, and Phenylacetaldehyde, were selected as the potential biomarkers for EIMD prediction. 2′-Deoxycytidine is a key component in DNA synthesis and participates in the DNA methylation process in epigenetic modifications, while Cytosine is the basic unit of nucleic acids. Phenylacetaldehyde is a metabolic intermediate of aromatic amino acids such as phenylalanine. Pyridoxamine is one of the active forms of vitamin B6. All these metabolites have not been reported in previous studies on exercise or as the biomarkers of EIMD. Future studies will focus on the development of the EIMD diagnosis method by detecting these metabolites.

Although this study provides potential urinary biomarkers for EIMD, thereby advancing the development of non-invasive diagnostic methods, several limitations should be acknowledged. Primarily, the data used in this study were all collected immediately post-exercise. While CK typically peaks between 24 and 72 h, frequent measurements are often difficult for participants to tolerate. To address this, we conducted a VAS test, which has been shown to have a certain extent of correlation with CK [48]. Future research should further validate the relationship between these metabolites and subsequent CK kinetics, particularly in the context of different genders, sports, and individual training levels. Additionally, due to the limited number of participants, only male participants were recruited to avoid sex differences. Future studies will focus on the metabolite changes in female participants. Finally, integrating more comprehensive transcriptomic and proteomic technologies could help elucidate the dynamic changes in urinary metabolite profiles associated with EIMD, immune response, and oxidative stress, thereby providing deeper insights into this research topic.

## 5. Conclusions

AIRT induced EIMD in all participants and significantly altered urinary metabolite profiles. The changes in urinary metabolites and pathways were due to the metabolic adaptation to oxidative stress, inflammatory responses, and ammonia metabolism imbalance. The selected four potential urinary biomarkers provide important evidence for the further development of a non-invasive, urine-based method for the immediate assessment of EIMD.

## Figures and Tables

**Table 1:** Basic characteristics of the subjects (n = 22).

Characteristics	Mean	Standard Deviation (SD)
Age	22	1.72
Resting Heart Rate	68.64	5.73
Height (cm)	178.64	4.98
Weight (kg)	76.24	10.47
Body Fat Percentage (%)	22.03	4.77
Body Mass Index	23.83	2.46
Muscle Mass (kg)	55.86	6.06

**Table 2:** Biomarkers Selected Based on Correlation Analysis Combined with VIP Values.

	Metabolite Name	VIP	R
CK	Pyridoxamine	1.054	−0.407
	cytosine	2.122	−0.403
LDH	cytosine	2.122	−0.709
	Pyridoxamine	1.054	−0.600
	Phenylacetaldehyde	1.077	−0.573
	2′-Deoxycytidine	2.470	0.303

## Data Availability

The original contributions presented in this study are included in the article. Further inquiries can be directed to the corresponding author.

## Abbreviations

The following abbreviations are used in this manuscript:

AIRT	Acute Intermittent Rowing Training
AUC	Area Under the Curve
CK	Creatine Kinase
CK-MB	Creatine Kinase-MB Isoenzyme
CMJ	Countermovement Jump
DOMS	Delayed Onset Muscle Soreness
EIMD	Exercise-Induced Muscle Damage
HBDH	Hydroxybutyrate Dehydrogenase
HIIT	High-Intensity Interval Training
LDH	Lactate Dehydrogenase
PCA	Principal Component Analysis
PC	Principal Component
OPLS-DA	Orthogonal Partial Least Squares Discriminant Analysis
R	Correlation Coefficients
ROC	Receiver Operating Characteristic
VIP	Variable Importance in Projection
